# Children’s Health/Regional Collaboration to Reduce Lead Exposure in Children

**DOI:** 10.1289/ehp.115-1797867

**Published:** 2007-01

**Authors:** Mary Jean Brown, Henry Falk, Howard Frumkin

**Affiliations:** Division of Emergency and Environmental Health Services, National Center for Environmental Health Centers for Disease Control and Prevention Atlanta, Georgia, E-mail: mjb5@cdc.gov; Coordinating Center for Environmental, Health and Injury Prevention, Centers for Disease Control and Prevention, Atlanta, Georgia; National Center for Environmental Health, Agency for Toxic Substances and Disease, Registry, Centers for Disease Control and Prevention, Atlanta, Georgia

As Safi et al. (2006) discussed, environmental contamination does not stop at international boundaries. An excellent example of a collaborative effort to address regional environmental exposures is that of the public health communities in Israel, Jordan, and the Palestinian Authority to assess and limit lead exposure of young children. Their dedication to this project in the face of significant political upheavals and episodic violence has demonstrated a remarkable commitment among international public health colleagues to improve environmental public health.

Safi et al. (2006) underscored the three most important strategies to prevent lead exposure in young children. First, eliminate leaded gasoline. In countries where this strategy has been successfully implemented, blood lead levels have significantly decreased (Pirkle et al. 1994; Schnass et al. 2004). More than 50 nations have eliminated lead in gasoline, and many others will initiate phase-outs over the next few years (Landrigan 2002).

Second, identify other consequential sources of lead and take action to control or eliminate them. Smelting remains a prevalent hazard in many parts of the world (ATSDR 1999). Efforts such as recycling batteries in controlled facilities have been successful in some countries.

Third, expand surveillance to ensure that recurrent or new sources of lead exposure are identified and that appropriate actions are taken. Both children and exposure sources travel. In the United States, we have found that the risk of lead exposure is much higher among immigrants when they arrive in the United States, usually as a result of use of lead-containing products; this elevated risk for exposure continues after immigrants relocate when the children are exposed to lead in paint and house dust (CDC 2005).

This collaborative project in the Middle East is an outstanding model for other international efforts to control environmental contaminants in complex regional settings. Safi et al. (2006) have shown tremendous vision, integrity, and commitment to public health under very difficult circumstances.
